# Effects of *Clostridium difficile* Toxin A and B on Human T Lymphocyte Migration

**DOI:** 10.3390/toxins5050926

**Published:** 2013-05-03

**Authors:** Dan Wu, Antony George Joyee, Saravanan Nandagopal, Marianela Lopez, Xiuli Ma, Jody Berry, Francis Lin

**Affiliations:** 1 Department of Physics and Astronomy, University of Manitoba, Winnipeg, MB R3T 2N2, Canada; E-Mails: dan@physics.umanitoba.ca (D.W.); sarans@physics.umanitoba.ca (S.N.); xlma79@yahoo.com (X.M.); 2 Cangene Corporation, Winnipeg, MB R3T 2N2, Canada; E-Mails: jageorge@cangene.com (A.G.J.); jody_berry@bd.com (J.B.); 3 Department of Immunology, University of Manitoba, Winnipeg, MB R3E 0T5, Canada; 4 Department of Biosystems Engineering, University of Manitoba, Winnipeg, MB R3T 2N2, Canada; 5 Department of Medical Microbiology, University of Manitoba, Winnipeg, MB R3E 0J9, Canada; E-Mail: amarianelalopez@gmail.com; 6 BD Biosciences, La Jolla, CA 92121, USA; 7 Department of Biological Sciences, University of Manitoba, Winnipeg, MB R3T 2N2, Canada

**Keywords:** *C. difficile* toxin A and B, human T lymphocyte, cell migration, chemotaxis, microfluidic device

## Abstract

Bacterial products such as toxins can interfere with a variety of cellular processes, leading to severe human diseases. *Clostridium difficile* toxins, TcdA and TcdB are the primary contributing factors to the pathogenesis of *C. difficile*-associated diseases (CDAD). While the mechanisms for TcdA and TcdB mediated cellular responses are complex, it has been shown that these toxins can alter chemotactic responses of neutrophils and intestinal epithelial cells leading to innate immune responses and tissue damages. The effects of *C. difficile* toxins on the migration and trafficking of other leukocyte subsets, such as T lymphocytes, are not clear and may have potential implications for adaptive immunity. We investigated here the direct and indirect effects of TcdA and TcdB on the migration of human blood T cells using conventional cell migration assays and microfluidic devices. It has been found that, although both toxins decrease T cell motility, only TcdA but not TcdB decreases T cell chemotaxis. Similar effects are observed in T cell migration toward the TcdA- or TcdB-treated human epithelial cells. Our study demonstrated the primary role of TcdA (compared to TcdB) in altering T cell migration and chemotaxis, suggesting possible implications for *C. difficile* toxin mediated adaptive immune responses in CDAD.

## 1. Introduction

*Clostridium difficile* (*C. difficile*) is a bacterial pathogen and a leading cause of hospital-acquired diarrhea in Europe and North America and of other *C. difficile*-associated diseases (CDAD) [1,2,3]. Particularly, the occurrence of CDAD has been consistently rising over the past few decades [1,4], in part associated with the increased usage of antibiotics as well as chemotherapies [1]. *C. difficile* produces two toxins, TcdA and TcdB, which are large multi-domain carbohydrate-binding proteins and constitute the major virulence factors [1,5,6]. The importance of TcdA and TcdB as the primary diagnostic markers for CDAD [1] has motivated the health research community to look into the underlying cellular mechanisms. It has been shown that TcdA and TcdB can cause intense intestinal inflammation and tissue damage [7]. Such effects are possibly resulting from the known TcdA and TcdB mediated cell cytoskeleton disorganization and cell death [8,9]. Indeed, TcdA induces impaired migration of intestinal epithelial cells in a dose-dependent manner as well as apoptosis of various cell types such as epithelial cells, endothelial cells and monocytes [10,11,12,13,14,15,16,17]. In addition to the direct inhibition of cell migration and the killing effects, exposure of intestinal epithelial cells to TcdA leads to up-regulated chemokine production, which can cause increased neutrophil infiltration to intestinal tissues [1,18]. Furthermore, TcdA can directly mediate neutrophil recruitment to the infection site during pseudomembranous colitis as studied in rabbit models [19]. Although TcdB has similar effects on intestinal tissue disruption and apoptosis of epithelial cells [20], its ability for altering the migratory responses of relevant cell types is not defined.

Taken together the current knowledge of TcdA and TcdB mediated cellular responses, it is tempting to hypothesize that TcdA and TcdB are able to affect cellular behaviors, particularly the migration and trafficking of other important immune cell types such as T lymphocytes. Such a hypothesis is further supported by previous studies showing that lethal and edema toxins of *Bacillus anthracis* affect the activation and cellular functions of dendritic cells and lymphocytes [21]. Particularly, both anthrax toxins suppress T cell chemotaxis towards the homeostatic chemokine, SDF-1α, by inhibiting the activation of MAP kinases in a chemokine receptor dependent manner [21,22]. On the other hand, it has been shown that the production of lymphocyte relevant chemoattractants by the intestinal epithelial cells is not affected upon exposure to TcdA [18]. Thus, it is interesting and important to address the questions: (1) Can TcdA and TcdB alter T cell migration and chemotaxis? (2) Can these effects be induced in a direct and/or indirect manner? (3) Do TcdA and TcdB have similar or differential effects on T cell migration and chemotaxis? Answers to these questions will provide important scientific basis for linking *C. difficile* toxins and adaptive immune response with the potential for developing new strategies of treating or preventing CDAD. To address these questions, we investigated the direct and indirect effects of TcdA and TcdB on the migration and chemotaxis of human blood T cells using both conventional transwell cell migration assays and a microfluidic device that allows quantitative single cell migration analysis in well-defined chemokine gradient environments [23,24,25].

## 2. Results and Discussion

### 2.1. Effects of TcdA and TcdB on T Cell Viability

**Figure 1:**
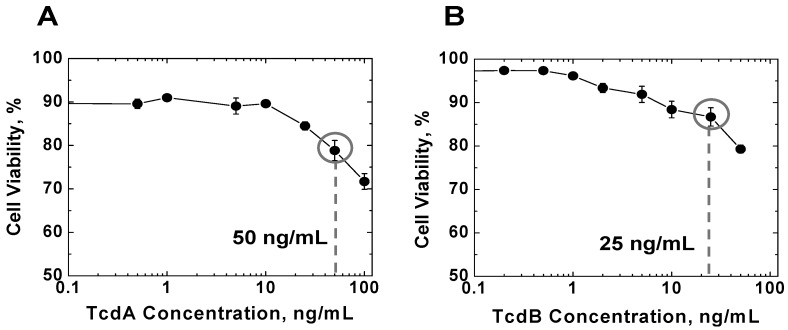
Effects of TcdA and TcdB on T cell viability. Human blood T cells were treated with TcdA (**A**) or TcdB (**B**) for 3 h and the cell viability was determined by trypan blue staining using a hemocytometer (at least 3 independent experiments for each condition). The circled toxin concentrations (*i.e.*, TcdA: 50 ng/mL; TcdB: 25 ng/mL) were selected for all subsequent experiments. (*i.e.*, the qualitative selection criteria for TcdA and TcdB concentration are (1) comparable viability level ~80%; (2) the concentration before the viability level drops relatively fast.).

In order to investigate the role of TcdA and TcdB in killing and migration of human blood T cells, initially we tested several concentrations of these toxins. T cells were incubated with a range of concentrations of TcdA and TcdB for 3 h in T cell culture medium in well plates inside a 37 °C incubator with 8% CO_2_ injection. Then cell viability was measured by the standard trypan blue staining using a hemocytometer. As we expected, the viability of T cells decreased with the increasing concentration of TcdA or TcdB (Figure 1). Thus, TcdA and TcdB have a toxic effect on human blood T cells. To maximize the possible effects of TcdA and TcdB on T cell migration without significant complications from the killing effect, we selected 50 ng/mL TcdA and 25 ng/mL TcdB based on the measured killing curves for all subsequent experiments (Figure 1) (*i.e.*, the qualitative selection criteria for TcdA and TcdB concentration are (1) comparable viability level ~80%; (2) the concentration before the viability level drops relatively fast.) 

### 2.2. Effects of TcdA and TcdB on T Cell Motility Measured by Transwell Assays

**Figure 2:**
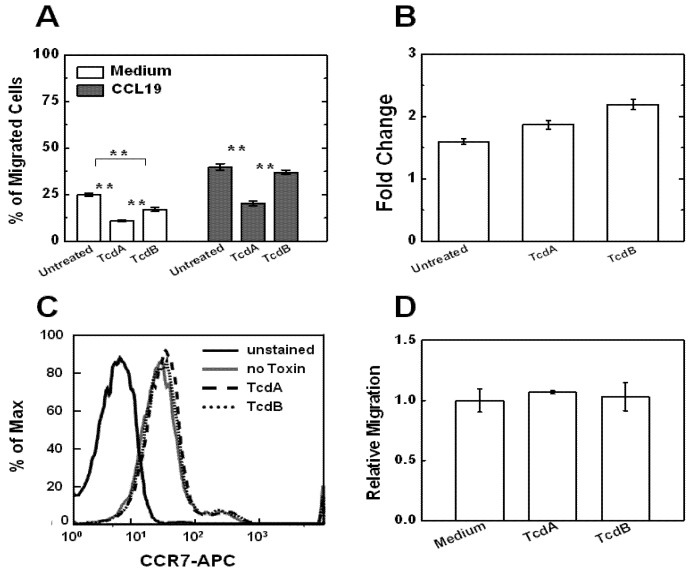
Effects of TcdA and TcdB on T cell motility and chemotaxis in transwell assays. (**A**) Migration of TcdA- or TcdB pre-treated T cells or untreated cells to either medium alone or medium containing CCL19 (100 nM), is presented as the percentage of input cells that migrated to the bottom well of the transwell assay; (**B**) Fold change of cell migration to 100 nM CCL19 comparing to medium alone; (**C**) Flowcytometric analysis of CCR7 expression on T cells with or without TcdA or TcdB treatment. T cells were incubated with 50 ng/mL TcdA or 25 ng/mL TcdB for 3 h before CCR7 antibody staining (anti-human CCR7-APC); (**D**) T cell migration to the medium control, 50 ng/mL TcdA or 25 ng/mL TcdB. Data are normalized to the percentage of T cells migrated to medium alone. All migration experiments (at least 3 independent experiments for each condition) were performed in RPMI containing 0.4% BSA for 90 min. The *p* values for each comparison from the 2-sample *t* test are shown: ** *p <* 0.01.

Upon pre-treatment of TcdA or TcdB at the doses as described in the previous section, T cell motility was tested using the conventional transwell assay. Our results showed significant decrease of T cell motility upon TcdA or TcdB treatment as quantified by the percentage of cells migrating to the bottom well in the absence of any chemoattractant stimulation (Figure 2A). 

### 2.3. Effects of TcdA and TcdB on T Cell Chemotaxis

Next, we tested the effect of TcdA or TcdB pre-treatment on T cell chemotaxis to a chemokine gradient. Interestingly, our results from transwell assays showed that TcdA but not TcdB pre-treatment significantly decreases T cell chemotaxis to a 100 nM CCL19 gradient (Figure 2A). Further analyzing the fold change of cell migration suggested that the decreased motility of TcdA- or TcdB-treated T cells recovers upon chemokine gradient stimulation (Figure 2B).

To test if TcdA and TcdB alters T cell motility and chemotaxis by modifying the expression level of CCR7 (the chemokine receptor for CCL19), we measured CCR7 expression in T cells with or without TcdA or TcdB treatment by flow cytometry. Our results indicated that TcdA or TcdB does not affect CCR7 expression (Figure 2C) and thus we excluded this simple CCR7-dependent mechanism. We further confirmed that TcdA or TcdB by themselves do not attract or repel T cells (Figure 2D) in the transwell assay. Thus, these results suggest that TcdA and TcdB pre-treatment can impair the basal motility of T cells in the absence of other guiding or co-stimulatory signals.

**Figure 3:**
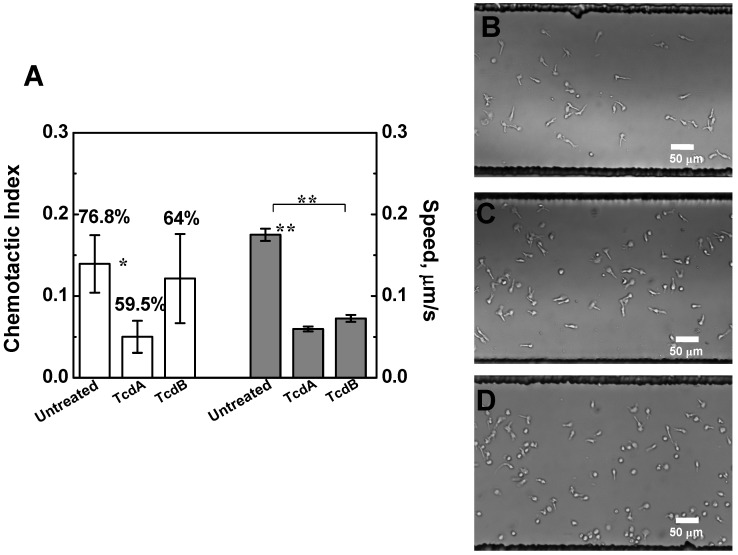
Effects of TcdA and TcdB on T cell chemotaxis to a CCL19 gradient in microfluidic devices. (**A**) Chemotactic Index (C.I.) and speed of T cells with or without toxin pre-treatment (TcdA: 50 ng/mL; TcdB: 25 ng/mL; 3 h treatment before the cell migration experiments) over a 35 min cell migration experiment in a 100 nM CCL19 gradient. Results are presented as average ± S.E.M. The percentage of cells migrating towards the CCL19 gradient is shown on the top of the C.I; (**B**–**D**) Images of T cells without toxin treatment (**B**), treated by TcdA (**C**) or TcdB (**D**) in microfluidic devices. The *p* values for each comparison from the 2-sample *t* test are shown. * *p* < 0.05; ** *p* < 0.01. Three independent experiments were performed for each condition with similar results and one representative experiment for each condition is presented.

Because of the unique ability of microfluidic devices of generating stable chemokine gradients with well-defined gradient profiles and of dynamic monitoring and quantitative analysis of cell migration at the single cell level, we further studied T cell chemotaxis using a previously developed microfluidic device [23]. Our results confirmed the differential effect of TcdA and TcdB on T cell chemotaxis to the CCL19 gradient (Figure 3A). Specifically, T cells treated with TcdA but not TcdB significantly decreased their chemotactic orientation toward the 100 nM CCL19 gradient whereas the cell speed consistently decreased with TcdA or TcdB treatment (Figure 3A). No apparent morphology changes were observed in T cells treated with TcdA or TcdB (Figure 3B–D). Taken together the results from both transwell assays and microfluidic devices, we conclude that TcdA but not TcdB decreases human blood T cell chemotaxis to a CCL19 gradient in the current specific experimental setting.

### 2.4. Effects of TcdA and TcdB on T Cell Migration to HT-29 Cell Culture

**Figure 4:**
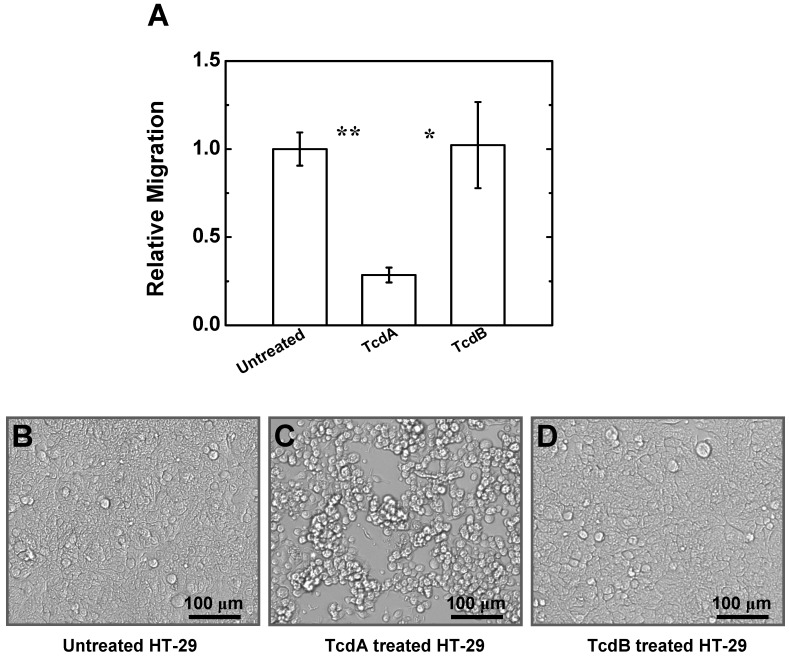
Effects of TcdA or TcdB on T cell migration to HT-29 cell culture. (**A**) T cell migration to HT-29 cell culture treated with TcdA or TcdB for 24 h or to untreated HT-29 cell culture. Data are normalized to the percentage of T cells migrated to the untreated HT-29 cell culture; (**B**–**D**) Morphological changes of HT-29 cells without (**B**) or with TcdA (**C**) or TcdB (**D**) treatment after 24 h. All cell migration experiment were performed in RPMI with 10% FBS for 90 min. The *p* values for each comparison from the 2-sample *t* test are shown. * *p* < 0.05; ** *p* < 0.01.

Because TcdA and TcdB can interact with intestinal epithelial cells to mediate neutrophil infiltration to intestinal tissues [1], it is possible that TcdA and TcdB can also alter T cell migration indirectly through intestinal epithelial cells. Thus, we performed transwell assays to examine human T cell migration to the HT-29 cells (a human intestinal epithelial cell line) with or without TcdA or TcdB treatment. In the transwell experiments, HT-29 cells were incubated with TcdA or TcdB for 24 h and then T cell migration to HT-29 cell culture was assessed. Our results showed that T cell migration to the TcdA-treated HT-29 cell culture was significantly lower comparing to the untreated HT-29 cell culture (Figure 4A). By contrast, T cell migration to the TcdB-treated HT-29 cell culture was not affected (Figure 4A). Such a difference is consistent with the rounding and shrinking of TcdA-treated HT-29 cells and thus, a disruption of cell monolayers compared to the untreated or TcdB-treated cells (Figure 4B–D). This set of data further confirmed the differential effects of TcdA and TcdB on T cell migration.

## 3. Experimental Section

### 3.1. Reagents

Recombinant TcdA and TcdB were prepared and provided by Cangene Corporation. Human recombinant CCL19, anti-human CD3/CD28 antibodies were purchased from Cedarlane (Burlington, ON, Canada). Human recombinant IL-2 was obtained from NCI-Frederick. Histopaque 1077 was purchased from Sigma Aldrich (St. Louis, MO, USA). All other reagents were purchased through Fisher Scientific (Ottawa, ON, Canada).

### 3.2. Cells

Human peripheral blood samples were collected from healthy adult donors in collaboration with The Victoria General Hospital at Winnipeg with an approved human ethics protocol. Peripheral blood mononuclear cells (PBMC) were isolated using the standard gradient centrifugation method. T cells from total PBMC were activated by incubation with anti-CD3/CD28 antibodies for 2 days in culture medium (RPMI-1640 with 10% FBS) in a 37 °C incubator with 8% CO_2_ injection. Activated T cells were then expanded in the presence of IL-2 and were cultured for at least 4 days before cell migration experiments. The human intestinal epithelial cell line HT-29 cells were provided by Cangene Corporation and were cultured in McCoy’s 5a medium supplemented with 10% FBS in a 37 °C incubator with 5% CO_2_ injection, and were passaged regularly for the use of specific experiments throughout this study.

### 3.3. Flow Cytometric Analysis of Surface CCR7 Expression

T cells with or without TcdA or TcdB treatment were stained with APC-conjugated anti-human CCR7 antibody (Cedarlane, Burlington, ON, Canada), followed by flow cytometric analysis of surface CCR7 expression using a flow cytometer (BD FACSCalibur, Becton Dickinson, Mississauga, ON, Canada). The flow cytometry data were further analyzed using Flow Jo (Tree Star, Inc., Ashland, OR, USA).

### 3.4. Transwell Cell Migration Assays

Chemotaxis of T cells to CCL19, TcdA, TcdB and HT-29 cell culture (with or without TcdA or TcdB treatment) was tested using transwell chemotaxis assays (COSTAR 24 well plate with inserts of 5 μm pore, Corning, NY, USA) [24]. Briefly, migration medium (RPMI-1640 with 0.4% BSA) with or without chemokines of defined concentrations was added to the bottom well in a 600 μL volume. Then 10^6^ cells in 100 μL migration medium were added to the top well. Cell migration was assayed for 1.5 h in a 37 °C incubator with 8% CO_2_ injection. Then the inserts were carefully removed, and the cells that had migrated through the insert to the bottom well were counted using a flow cytometer (BD FACSCalibur, Becton Dickinson, Mississauga, ON, Canada). 15 μm diameter calibration beads (Polysciences, Inc., Warrington, PA, USA) were mixed with the cell samples as an internal control for accurate cell counting by flow cytometry. All experiments were performed in triplicate. Chemotaxis of cells is measured as the percentage of the input cells that migrated to the bottom well. The FACS data was further analyzed using Flow Jo (Tree Star, Inc., Ashland, OR, USA). Two-sample *t*-test was performed to determine the statistical significance for comparing different conditions.

### 3.5. Microfluidic Device and Gradient Generation

A previously reported “Y” shape microfluidic device was used for single cell migration experiments [23]. The microfluidic device was designed in Freehand 9.0 (Adobe Systems Inc., San Jose, CA, USA) and the design was printed to a transparency mask by a high resolution printer. The masters were fabricated at The Nano Systems Fabrication Laboratory (NSFL) at the University of Manitoba. The design was patterned on a silicon wafer by contact photolithography with SU-8 photoresist (Micro Chem, Newton, MA, USA) through the transparency mask and the SU-8 pattern yields ~100 µm thickness. The polydimethylsiloxane (PDMS) replicas were then fabricated by molding PDMS (Sylgard 184 silicon elastomer, Dow Corning, MI, USA) against the master. Two 1 mm diameter holes for the two fluidic inlets and one 4 mm diameter hole for the fluidic outlet were punched out of the PDMS replica respectively using sharpened needles. An additional 1 mm hole was punched for loading cells. The PDMS replica was then plasma bonded to a glass slide using a plasma cleaner (Harrick Plasma, Ithaca, NY, USA). Polyethylene tubing (PE-20, Becton Dickinson, Mississauga, ON, Canada) was inserted into the inlet holes to connect the microfluidic device to syringe pumps (KD Scientific, Holliston, MA, USA) with two 250 µL syringes containing medium or chemokine solutions for fluidic infusion. Chemokine solutions of defined concentration were prepared in migration medium (RPMI-1640 with 0.4% BSA). FITC-Dextran 10 kDa that has similar molecular weight of the chemokine molecule was added to the chemokine solution. The migration medium and chemokine solutions were continuously infused into the device by syringe pumps through tubing and the inlets of the device at the total flow rate of 0.2 µL/min for generating stable chemokine gradient in the main microfluidic channel. The gradient was confirmed by measuring the fluorescence intensity profile of FITC-Dextran at ~3mm downstream of the “Y” junction inside the microfluidic channel where the gradient yields a smooth profile. This region was then chosen for imaging cells in the subsequent cell migration experiments.

### 3.6. Cell Migration Experiments Using Microfluidic Device

The microfluidic channel was coated with fibronectin (BD Biosciences, Mississauga, ON, Canada) for 1 h at room temperature and blocked with BSA for another hour before the experiment. For each experiment, cells were loaded into the microfluidic device from the cell inlet and allowed to settle in the fibronectin-coated channel for ~5 min. The device was maintained at 37 °C by attaching a transparent heater to the back of the cover slide (Thermal-Clear Transparent Heater, Model No. H15227, Minco, Minneapolis, MN, USA). The heater was powered by a DC power supply (Model No. 6204A, Harrison, NY, Canada) and was controlled by a sensorless temperature controller (Model No. CT198, Minco, Minneapolis, MN, USA). The temperature was calibrated to 37 °C using a digital thermometer (VWR, Mississauga, ON, Canada). Medium and chemokine solutions were infused into the device to apply the chemokine gradient to cells. The device was placed on a microscope (Olympus BX60, Olympus Canada Inc., Richmond Hill, ON, Canada). The system was allowed to equilibrate for ~5 min (wait until no flowing cells were seen in the channel) and cell migration was recorded by time-lapse microscopy at 6 frames/min for 35 min using a CCD camera (Model No. 370 KL 1044, Optikon, Kitchener, ON, Canada). The image acquisition was controlled by NIH ImageJ (v.1.45n). 

### 3.7. Analysis of Microfluidic-Based Cell Migration Data

Movement of individual cells in the microfluidic device was tracked using MetaMorph (Offline Premier Version, v7.7.3, Molecular Devices Inc., Sunnyvale, CA, USA). The images were calibrated to distance and only the cells that migrated within the field of view were selected and tracked. The tracking data were exported to Excel and MATLAB for analysis. Following previously established analysis methods [23,24,25], the movement of cells was quantitatively evaluated by (A) the percentage of cells that migrated toward the chemokine gradient; (B) the Chemotactic Index (C.I.), which is the ratio of the displacement of cells toward the chemokine gradient to the total migration distance, presented as the average value ± standard error of the mean (S.E.M); (C) the speed of cells, which is the total migration distance divided by the migration time, presented as the average value ± S.E.M; The parameters between different conditions were compared by the 2 sample *t* test. 36–89 cells were analyzed for each experiment.

## 4. Discussion and Conclusions

Immune cell migration and homing are key players in the outcome of immune responses and are finely tuned by chemokines [26,27]. Naïve T lymphocytes migrate from the bloodstream to lymphoid organs, where they interact with antigen presenting cells (APCs), which have migrated from the site of infection after uptake of the pathogen or its secreted products. Once T-cells have encountered an APC bearing MHC bound cognate antigen, they stop, become activated, proliferate and differentiate to armed effectors. Effector T cells then exit the lymphoid organs and patrol the body in search of the site where the invading pathogen is located. In response to certain chemokines, T cells migrate to the inflamed tissue and cooperate with the phagocytes to clear the infection. Thus, chemokine gradients are crucial for regulating immune cell trafficking [27,28,29].

Our study focused on the effects of *C. difficile* TcdA and TcdB on T cell migration and chemotaxis to chemokine gradients. For the initial study, we targeted chemokine CCL19 and its receptor CCR7 in activated human peripheral blood T cells. A high percentage of activated human peripheral blood T cells express surface CCR7 as shown in previous studies [23] and in the present study (Figure 2C). CCL19 is a potent chemoattractant for T cells *in vitro* and a key player for mediating T cell trafficking *in vivo* such as in secondary lymphoid tissues [30,31,32,33]. In addition, CCL19 mediates robust chemotactic migration of activated human peripheral blood T cells in both traditional transwell assays and microfluidic devices [23]. In contrast to CCL21, the other ligand for CCR7, which can form gradients in both soluble and immobilized form in tissues and other extracellular matrices (ECM), CCL19 gradients are presented only in soluble form [34]. Therefore, CCL19 is a reasonable choice as the initial chemokine for the present study due to its physiological importance and it is relatively simple and practical for T cell migration analysis in our experimental systems.

The transwell assays allow fast testing of cell migration in different conditions at the cell population level. However, such an assay has the limitation in chemical gradient control and does not allow real time visualization of cell movement. By contrast, microfluidic devices can precisely control the gradient conditions and allow quantitative cell migration analysis at the single cell level although the throughput of the current microfluidic chemotaxis device is still low. Thus, combining the use of transwell assays and microfluidic devices allowed us to obtain complementary data for the effects of TcdA and TcdB on T cell migration and chemtoaxis. Quantitative cell migration data analysis from microfluidic experiments showed significant decrease of cell speed following treatment with either toxin (Figure 3A). Therefore, both TcdA and TcdB may impair T cell migration and chemotaxis at the motility level. The unaffected CCR7 expression level in T cells with TcdA or TcdB treatment suggested that the TcdA or TcdB induced alteration of T cell migration and chemotaxis in the CCL19 gradient is not through simple CCR7 down-regulation and thus further research is required to explore its underlying mechanism.

In the present study, we used activated human peripheral blood T cells for the chemotaxis experiments to CCL19. From our experience, activated T cells migrate considerably better than naïve T cells in microfluidic devices. In addition, our previous study suggested that CCL19 and CCL21 play interesting roles in mediating the migration and trafficking of activated T cells in sub-regions of lymph nodes [25]. These considerations led to the selection of activated T cells for chemotaxis experiments in this study. The observed decrease of chemotaxis of TcdA- but not TcdB-treated T cells to CCL19 suggested that TcdA, but not TcdB may directly interfere with T cells trafficking in secondary lymphoid tissues and thus alter adaptive immune responses after toxin exposure. In the future, it will be important to further test the effects of TcdA and TcdB on the migration and chemotaxis of naïve T cells to CCL19. In addition, further studies to test the effects of TcdA and TcdB on T cell chemotaxis to other chemokines (e.g., CCL21, a second ligand for CCR7; CCL25, the chemokine that regulates T cell homing to intestinal tissues) using the experimental approach developed in this study will provide more insights into TcdA and TcdB mediated T cell migration and trafficking in specific relevant tissues.

Motivated by the previous studies showing that *C. difficile* toxin exposed intestinal epithelial cells mediate neutrophil infiltration to the intestinal tissues [1], we tested if TcdA- or TcdB-treated intestinal epithelial cells may have similar effects on T cell migration. To our surprise, we found that T cell migration to HT-29 cell culture was either decreased or unaffected by treatment with TcdA or TcdB respectively. Further studies are required to identify the effecting factors (possibly secreted by TcdA-exposed intestinal cells) that weaken T cell migration. The differential effects of TcdA and TcdB on T cell migration to HT-29 cell culture once again suggested the differential ability of these two toxins for mediating T cell migration and trafficking. Although both TcdA and TcdB are expected to affect HT-29 cell viability [20,35], their differential effect on HL-29 cell morphology (Figure 4C,D), and thus possibly on cell viability as well, may be linked to the observed differential migratory response of T cells to HT-29 cell culture with TcdA or TcdB treatment (Figure 4A).

Thus, by inhibiting migration of T cells, *C. difficile* can hamper the initiation of adaptive immune responses, including B cell differentiation and thus, impairing production of protective antibodies. Indeed, a study [36] showed that asymptomatic carriers of *C. difficile* had significantly higher serum IgG antibody levels to TcdA compared with colonized patients who later developed diarrhea. Our study examined and showed for the first time the effects of TcdA and TcdB on human T cell migration and chemotaxis. The results from transwell assays and microfluidics-based cell migration analysis showed interesting differential effects of TcdA and TcdB in altering T cell migration and chemotaxis in either a direct or indirect manner. These findings suggested the possible roles played by *C. difficile* toxins in mediating T cell migration and trafficking with relevance for the pathology of CDAD. Further studies to better understand the underlying cellular mechanisms for toxin-T cell interactions may provide the scientific basis for developing new therapeutic approaches or prevention strategies for CDAD.
